# Rapid Synthesis of Thiol-Co-Capped-CdTe/CdSe/ZnSe Core Shell-Shell Nanoparticles: Their Optical and Structural Morphology

**DOI:** 10.3390/nano11051193

**Published:** 2021-05-01

**Authors:** Olamide Abiodun Daramola, Xavier Siwe-Noundou, Potlaki Foster Tseki, Rui Werner Maçedo Krause

**Affiliations:** 1 Department of Chemistry, Faculty of Science, Rhodes University, P.O. Box 94, Grahamstown 6140, South Africa; truelamm@gmail.com; 2 Department of Chemical and Physical Sciences, Faculty of Natural Science, Walter Sisulu University, Private Bag XI, Mthatha 5117, South Africa; ptseki@wsu.ac.za

**Keywords:** thiol dual-capped, CdTe multi-core-shell, rapid synthesis

## Abstract

CdTe QDs has been demonstrated in many studies to possess good outstanding optical and photo-physical properties. However, it has been established from literature that the toxic Cd^2+^ that tends to leak out into nearby solutions can be protected by less toxic ZnS or ZnSe shells leading to the synthesis of core-shells and multi-core-shells. Hence, this has allowed the synthesis of CdTe multi-core-shells to have gained much interest. The preparation of most CdTe multi-core-shells reported from various studies usually has a longer reaction time (6–24 h) in reaching their highest emission maxima. The synthesis of CdTe multi-core-shells in this study only took 35 min to obtain a highest emission maximum compared to what has been reported from the literature. CdTe multi-core-shells were synthesized by injecting 7, 14, and 21 mL each of Zn complex solution and Se ions into the reacting mixture containing CdTe core-shells (3 h) at 5 min intervals over a 35 min reaction time. The emission maxima of the MPA-TGA-CdTe multi-core-shells at 21 mL injection was recorded around 625 nm. Therefore, we are reporting the rapid synthesis of five different thiol co-capped CdTe/CdSe/ZnSe multi-core-shell QDs with the highest emission maxima obtained at 35 min reaction time.

## 1. Introduction

Quantum dots (QDs) are ideal candidates for theranostics platforms due to their tremendous optical and photo-physical properties [1,2,3]. The quantum dots possess characteristics such as good chemical and photo-stability, high quantum yield and size, and tunable light emission [4,5,6]. These physical properties allow them to emit light from blue to near infra-red wavelengths (>650 nm) comparable to organic dyes. In addition, their characteristic physical properties place them as suitable diagnostic modalities in various clinical processes including deep tissue imaging [7,8,9,10,11]. A widely-used QD that has found application in diverse fields is the CdTe QDs. This is due to its outstanding optical properties compared to other similar QDs, such as CdS, CdSe, ZnS, and ZnSe [12,13,14,15].

The successful synthesis of CdTe multi-core-shell nanoparticles has engendered the production of material with high florescence intensity, long-term photo stability, and reduced toxicity. The transformation of the CdTe core to multicore-shells using ZnS or ZnSe as the outer shell tends to improve the bio-compatibility of the synthesized heterostructure by limiting the toxic Cd ion’s direct interaction with cells [16,17,18]. This affords the usage of the synthesized QDs as optical probes in various imaging studies including deep-tissue cellular imaging applications.

As a specific example, in 2011 water soluble and near-infrared emitting CdTe/CdSe/ZnSe quantum dots were synthesized in a simple one-pot procedure by Taniguchi and co-workers; these QDs were used in studies of subcutaneous deep tissue monitoring of Wister rats [17]. Green and co-worker also passivated the surface of CdTe/CdS core-shells with a ZnS shell using a simple successive injection of precursors in a one-pot reaction producing CdTe/CdS/ZnS multi-core-shell nanocrystals (2009). The multi-core-shell QDs synthesized were successfully used in imaging HeLa cells with reduced cyto-toxicity compared to the bare CdTe/CdS core-shells [16]. Recently, eco-friendly synthesis of GSH-CdTe/CdSe/ZnSe double-shell QDs were reported by Monaheng and co-workers in 2019, their study was followed by an evaluation of cytotoxicity and genotoxicity effects of these heterostructures on Chinese hamster ovary cells [18]. Arginine-functionalized CdTe/CdSe/ZnSe nanoparticles were also synthesized under ambient conditions in the absence of an inert environment. The study was reported by Vuyelwa et al. in 2017 and it established that these synthesized fluorophores demonstrated high fluorescent intensity with enhanced cell viability compared to bare QDs [19]. It is reported in a number of recent literature sources that the common types of thiol ligands that are used to stabilize the surface of synthesized CdTe multi-core-shells are mercapto-propanoic acid (MPA), glutathione (GSH), thioglycolic acid (TGA), and L-cysteine [12,15], this being due to their ability to enhance the emission intensity, chemical functionality, colloidal stability, water solubility, and bio-compatibility of synthesized fluorophores in aqueous media. Hence, in the presence of these stabilizing agents, CdTe multi-core-shells also demonstrated similar unique characteristics as observed in the literature cited above. However, it becomes clear from the literature that synthesized CdTe multi-core-shell heterostructures, by far, make use of single thiol ligand (MPA, GSH, TGA, and L-cysteine) to passivate their surfaces [16,17,18,19]. The few and limited studies demonstrating the use of two different thiol-ligands relate to the fabrication of CdTe cores and CdTe core-shells [20,21]. One reason for using more than one thiol ligand is to further improve the surface chemistry of synthesized nanoparticles. Typical examples of such studies were conducted by Yu et al. in 2012 [20] and by Adegoke and Nyokong in 2013 [21], where the combination of thiol-glycolic acid (TGA) and glutathione led to an improvement in the optical properties of synthesized CdTe cores and CdTe/ZnS core-shell nanoparticles [20,21]. It has also been reported by Wang et al. in 2012 concerning the electrochemiluminescence (ECL) studies of the CdTe QDs that the addition of TGA as a co-stabilizing agent increased the sensitivity of the synthesized CdTe QDs towards the selective detection of Pb^2+^ in aqueous media [22].

In light of the foregoing reports concerning the use of dual ligands, it seems instructive to stabilize the surface of synthesized CdTe multi-core-shell QDs with dual thiol ligands in order to improve their surface chemistry. A crucial piece of information reported from the literatures is that the majority of the synthesized CdTe multi-core-shell QDs usually have to undergo longer reaction times in achieving their emission maxima. As an example, the fabrication of CdTe/CdSe/ZnSe multicore-shells reported by Green et al. in 2009 took about 24 h in reaching an emission maximum of 660 nm [16]. Similar studies have reported longer reaction times, for example, Taniguchi et al. showed in 2011 that the synthesis of CdTe/CdSe/ZnSe multi-core shells took about 16 h to complete [17]. In 2012, Zhang and co-workers reported a study on the controlled optical properties of water-soluble CdTe/CdS/ZnS quantum dots. The synthesized type II CdTe multi core-shell was found to demonstrate a highest emission wavelength of 610 nm and took a total of 720 min of reaction time [23]. This long reaction time has serious implications on the use of available resources and energy.

In an effort to synthesize high-quality CdTe multi-core-shell QDs and improve on the overall synthesis times reported in the literature, herein we report the fabrication of five thiol dual-capped CdTe/CdSe core-shells with a simultaneous rapid synthesis (5 min) of their corresponding multi-core-shell with a maximum time of 35 min. The synthesis of these QDs involves optimizing their reaction conditions by injecting a total of 7, 14, and 21 mL each of Zn and Se ions at 5 min intervals into the reacting mixture of CdTe cores following a 3 h reaction time for the cores. In this way, the emission maxima (625 nm) of MPA-TGA-CdTe multi-core-shells at 21 mL of Zn^2+^ and Se^2−^ injection (35 min) was recorded as the highest emission maxima. The observed emission wavelength is considerably higher compared to similar thiol dual-capped QDs at equal reaction times. Further investigation into the photo-physical properties and the structural morphology of the synthesized multi-color materials was achieved using standard spectroscopic measurements. It is pertinent to point out that this reaction does not involve the use of a complex ligands or vigorous reaction condition. The synthesized materials are dual stabilized by the following: mercapto-propanoic acid-thiol-glycolic (M-T), L-cysteine-thiol-glycolic acid (L-T), glutathione-mercapto-propanoic acid (G-M), glutathione-thiolglycolic acid (G-T), and L-cysteine-mercapto-propanoic acid (L-M). The morphological effects of using two different thiol ligands on the QDs were also investigated with the hope of improving the surface chemistry of the synthesized multi-core-shell QDs.

## 2. Materials and Methods

### 2.1. Chemicals

Cadmium acetate [Cd (CH_3_COO)_2_], zinc sulphate (ZnSO_4_), potassium tellurite (K_2_TeO_3_), glutathione (GSH), mercapto-propanoic acid (MPA), thiol glycolic acid (TGA), L-cysteine, sodium borohydride (NaBH4), sodium sulfite, and selenium powder were all purchased from Sigma-Aldrich (Neustadt, Germany). All the chemicals were used without further purification.

### 2.2. Equipment

The absorption spectra of the dual-capped CdTe QDs were run on a Perkin Elmer Lambda 12 UV–VIS spectrophotometer in the wavelength range 200–1200 nm (Perkin Elmer, South Africa). A Perkin Elmer LS 55 photoluminescence (PL) spectrophotometer was used to record the photoluminescence spectra of the materials at an excitation wavelength of 400 nm (Perkin Elmer, South Africa). The measurements were conducted at room temperature using a 10 mm path length quartz cells. All Fourier transform infrared (FT-IR) spectra were measured using a Perkin Elmer Spectrum One (FTIR) coupled to a universal attenuated total reflection (ATR) sampling accessory (Perkin Elmer, South Africa). A Jeol2100 TEM operating at 200 KV was used for transmission electron microscopy (Zeiss Libra, Germany). All the TEM analysis were done by dropping the dilute solution of each of the samples on carbon-coated copper grids and the solvent slowly allowed to evaporate at room temperature. The energy dispersive X–ray spectroscopy (EDX) of the samples were measured using Vegan Tescan EDX spectrometer (Incapental FET, Germany)**.** The exposure of the QDs to UV irradiation was performed at an excitation wavelength of 314 nm at 130 W with UV lamp from Lasec South Africa (pty) limited.

The photoluminescence quantum yield (PLQY) of CdTe cores, CdTe core-shells, and CdTe-core-shell-shells was measured according to the method described by Crosby and Demas in 1971. Rhodamine 6G was chosen as a reference standard (QY = 95%, in ethanol), The standard and samples were both diluted to an absorbance of 0.05 at their corresponding excitation wavelength and the same solution was used to measure their florescence. The expression for the calculation of quantum yield is given as Equation (1), which follows:

(1) $$Y_{u\;}=Y_{s\;}\times \frac{F_{u}\times A_{s}}{F_{s}\times A_{u}}\times {\left(\frac{n_{u}}{n_{s}}\right)}^{2}$$

In Equation (1), the subscripts sand u denote standard (rhodamine 6G) and test samples respectively, Y is quantum yield, F is the integrated emission peak area, A is the absorbance at excitation wavelength, and n represents the solvent refractive index [10].

### 2.3. Methodology

#### 2.3.1. Synthesis of Dual-Capped-CdTe/CdSe Core-Shells and CdTe/CdSe/ZnSe Multi-Core-Shells

The syntheses of CdTe core-shells were achieved using a method we reported in 2017. The corresponding CdTe core-shells were promoted to their multi-core-shells following a method reported by Zhang et al. in 2011 but with substantial modification [24]. However, we recently reported the synthesis, photo-physics, and structural investigations of their corresponding cores [3].

#### 2.3.2. Selenium Reduction

For the reduction of selenium, sodium sulfite (Na_2_SO_3_); (0.5 g, 3.9 mmol) was added to deionized water (50 mL) that contained selenium powder (0.1574 g, 1.99 mmol). The reaction was allowed to proceed for 6 h with stirring at 70 °C to form sodium seleno-sulfite (Na_2_SeSO_3_).

#### 2.3.3. Synthesis of Dual-Capped-CdTe/CdSe Core-Shells

Following 3 h of reflux time of corresponding cores of each dual-capped CdTe QDs, reduced selenium solution (5 mL) was added drop-wise to coat the surface of respective CdTe cores. The addition of the selenium changed the color of the solution gradually from green to red within a few seconds. Aliquots were taken at regular intervals to monitor the growth of the shell on the surface of the core. The surface property of the synthesized material was then modified using volumes of reduced selenium solution to give a mole ratio of Te:Se: 1:0.2 (*v/v*, 1 mL of selenium solution), 1:0.4 (*v/v*, 2 mL of selenium solution), 1:0.6 (*v/v*, 3 mL of selenium solution). 1:0.8 (*v/v*, 4 mL of selenium solution), and 1:1 (*v/v*, 5 mL of selenium solution), respectively. It must be noted that as the volume of Se ions injected into the solution increases, the reaction time also increases. Injection at 1–5 mL of Se ions varied the reaction time from 10–50 s, approximately.

Note: The synthesis of the core-shell nanoparticles was conducted at 3 h cores while 3 h core-shell was used to build a multi-core-shell in order to size tune the corresponding synthesized material towards the near infra-red (NIR) for imaging purposes and high emission intensities.

#### 2.3.4. Formation of Zn Complex Ions

Zn sulfate (0.28 g, 1 mmol) was weighed and dissolved in 50 mL of deionized water. This was followed by the addition of dual capping agent, MPA (49 µL, 1 mmol), TGA (30 µL, 1 mmol), L-cysteine (0.121 g, 1 mmol), and glutathione (0.15 g, 1 mmol) into the reaction medium producing M-T, L-T, L-M, G-T, and G-M dual caps. The pH of the solution was adjusted to 10.8 using 1 M NaOH with continued stirring leading to the formation of the Zn complex ions.

#### 2.3.5. Formation of ZnSe Shell on CdTe/CdSe Core-Shells

During the construction of CdTe/CdSe core shells, equal volumes of Zn and Se ions (1 mL) were gradually injected seven times at intervals of 5 min into the reacting vessels following 3 h of CdTe/CdSe reflux time. This led to a total of 7 mL per each of Zn and Se ions that were injected into the solutions over 35 min. Aliquots were taken at 5 min intervals following the injection to monitor the photo-physics of the core-shells. However, aliquots at the 3 h mark following reflux were taken prior to the first injection of the Zn/Se solutions in order to monitor any wavelength shift following the injection of the first set of solutions at 5 min.

The color of the reaction was observed to be continuously changing as the volumes of Zn and Se ion (1–7 mL) increased signifying a change in material composition of the system. The condition of the reaction was further optimized by separately increasing the volumes of the Se and Zn solutions from 1 mL to 2 mL, making a total of 14 mL (2 mL every 5 min). Subsequently the volume was changed to 3 mL in the final experiment to make a total of 21 mL (3 mL every 5 min) of Zn and Se ions in the reacting vessels, in an attempt to improve the photo-physical properties of synthesized materials.

Following the synthesis of the five-thiol dual cap CdTe cores, CdTe/CdSe core-shells, and CdTe/CdSe/ZnSe core-shell-shells described in the text, the estimated particle sizes derived from corresponding UV–VIS excitonic peaks are provided in Table 1, Table 2, Table 3 and Table 4 according to Equation (2):

D = (9.8127 × 10^−7^) λ^3^ − (1.7147 × 10^−3^) λ^2^ + 1.0064 λ − 194.84 (2)

where λ represent excitation wavelength [13].

## 3. Results

### 3.1. Photophysical Investigation of CdTe Core Shells

#### 3.1.1. UV and PL Spectra of Dual-Capped-CdTe/CdSe Core-Shells

Results from the photo-emission spectra of M-T dual-capped CdTe/CdSe core-shell QDs indicate that virtually all synthesized core-shells at various mole ratios demonstrated high fluorescence intensity at 3 h reaction time for varying Te/Se ratios yielding values of PLQY of 80% (1:0.2), 72% (1:0.4), 70% (1:0.6), 75% (1:0.8) and 73% (1:1) (see Supplementary Information, Figure S1A–E). However, the largest wavelength shifts between 3 h core and 50 s core-shell can be observed at 1:1 of Te/Se (see Supplementary Information, Figure S1E). This accounts for the reason why this particular mole ratio for the core-shell was chosen as the scaffold for the synthesis of a multi-core shell. In addition, the remaining four dual-capped CdTe core-shell; (G-M, G-T, L-T and L-M) showed their best photo-physical result at 1:0.2 of Te/Se ratio and for the 3 h reaction time giving values of PLQY of 43% (G-M), 52% (G-T), 67% (L-T) and L-M (47%) which were higher when compared to the other mole ratios at equal reaction times (see Supplementary Information, Figures S2–S5). Therefore, this reaction time and mole ratios were selected as the best reaction conditions to synthesize each of the various core-shells to a multi-core-shell so as to maintain their enhanced photo-physical properties as they approach the red region of the spectrum.

#### 3.1.2. UV and PL Spectra of MPA-TGA-CdTe/CdSe Core-Shell QDs at 1:1 of Te/Se

**Figure 1:**
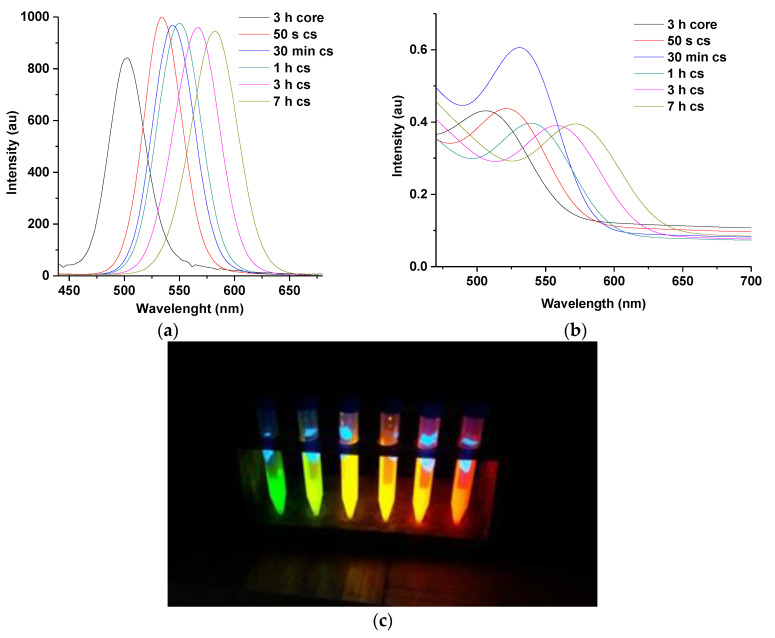
UV–VIS (**a**) and PL spectra (**b**) of MPA-TGA-CdTe/CdSe core shell at 1:1 of Te/Se and emission colors (**c**) produced at 3 h CdTe core (green)–7 h CdTe core-shell (orange). Note: cs represent core-shell.

The PL spectra in Figure 1a shows a significant shift of 34 nm in wavelength for CdTe core (496 nm at 3 h and for CdTe/CdSe core-shell (530 nm at 50 s), indicating an increase in particle size, within a short reaction time, due to the formation of CdSe shell at the surface of CdTe core. The construction of CdSe shell at the surface of the core is attributed to the reaction between Se ions injected into the solution and free Cd ions present inside the solution [19]. The concomitant increase in florescence intensity between 3 h CdTe cores (PLQY 65%), and 50 s CdTe core shells (PLQY 83%), with an increase in particle size indicating an increase in surface passivation provided by the thin layer of the CdSe shell at the surface of the core [13,23]. The thickness of the CdSe shell formed at the surface of the CdTe core was estimated to be about 0.2 nm. The corresponding particle size of CdTe cores at 3 h was calculated to be 2.7 nm and CdTe/CdSe core-shells at 50 s calculated to be 2.9 nm.

Furthermore, the passivation provided by the CdSe shell at the surface of the CdTe core is thought to reduce the surface defect of synthesized material thereby leading to an increase in emission intensity of the resulting CdTe core-shell. The emission intensity of the core-shell, however, decreased at 3 and 7 h of reaction time due to the formation of a thicker CdSe shell. Their estimated particle size according to Table 1 above is 3.4 and 3.5 nm, respectively, which would indicate an increase in shell growth compared to other reaction times [4,13].

Result from the UV spectra (Figure 1b) indicates a slight redshift in excitonic peak between 3 h for CdTe core and 50 s for CdTe core-shell due to the passivation of CdSe shell at the surface of CdTe core. It is observed that the intensity of the absorbance at 30 min reaction time is higher compared to others which might indicate enhanced surface re-construction process under open air condition as reaction time increases. However, the slight decrease in PL intensity after 50 s might be due to low surface re-combination effects by the charge carriers. However, further investigation is required to clarify this observation. Figure 1c indicates various emission colors produced by synthesized QDs under UV illumination. The green emission to the extreme left corresponds to 3 h for CdTe core which gradually changes to orange as one move from (L-R) due to the deposition of CdSe shell at the surface of CdTe core.

In Supplementary Information Figure S1A–E, it is observed that as the concentration of Se ion increases, significant shift in wavelength occurs at 3 h between the core and the core-shell corresponding to each mole ratio. The shift in wavelength seems to follow the order: 1:0.2 (4 nm), 1:0.4 (8 nm), 1:0.6 (16 nm), 1:0.8 (18 nm), 1:1 (36 nm) of Te/Se, respectively. The reason for the increase in wavelength shift is thought to be due to electro-static interaction between free Cd ions in the solution and reduced Se ions coming back into the solution. This would lead to formation of thicker CdSe shells thereby increasing the size of the heterostructure [2,4].

#### 3.1.3. PL Spectra of CdTe/CdSe Core-Shell QDs at 1:0.2 Dual-Capped with L-T, L-M, G-T, and G-M

It was mentioned earlier that CdTe core-shell dual-capped with L-T, L-M, G-T, and G-M indicate optimal fluorescence spectra for the Te/Se ratio at 1:0.2. This observation was ascribed to the optimal 3 h reaction time corresponding to this mole ratio.

**Figure 2:**
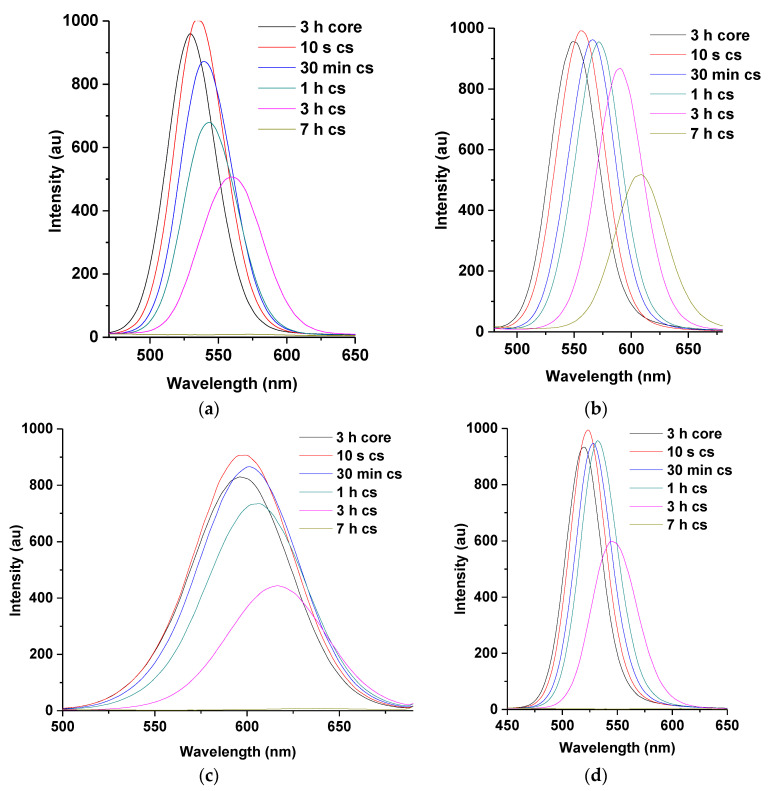
Typical PL spectra of L-M (**a**), L-T (**b**), G-M (**c**), and G-T (**d**) dual-capped CdTe/CdSe core-shell nanoparticles at 1:0.2 mole ratio of Te/Se for various reflux times (3 h CdTe core–7 h CdTe/CdSe core-shell). Note: cs represent core-shell.

All the dual-capped core-shell in Figure 2 demonstrate slight shift in wavelength between 3 h of their corresponding core to 10 s of their individual core-shell. An increase in emission intensity is also observed because of the passivation provided by the thin layer of the CdSe shell at the surface of the CdTe core [15,16,17]. This passivation could also account for the slight increase in emission wavelength between the two reaction times. The PLQY for L-M dual-capped was observed to increase from 68% for the core at 3 h to 87% for the core-shell at 10 s.

Furthermore, gradual decrease in fluorescence intensities of CdTe core-shell is observed from 30 min of reaction time. This is thought to be due to the formation of larger particle sizes at higher reaction time [1,19]. It is highlighted here that CdTe core-shells dual-capped with L-cysteine-thioglycolic acid (L-T) demonstrated the highest intensity of fluorescence at 3 h (PLQY 67%) and 7 h (PLQY 49%) reaction times among the dual-capped core-shells, as shown in Figure 2b. This observation is attributed to favorable surface chemistry interaction between L-cysteine and thioglycolic acid during the construction of the nanoparticle [3,7,12]. Hence, it is observed in all the spectra in Figure 2 that the combination of thio-glycolic acid (TGA) as dual stabilizing agents produces an improved surface passivation of synthesized CdTe core-shells. This is seen from the displayed intensities by G-T and L-T dual caps. The G-T dual-capped showed higher fluorescence intensities at 30 min (69%) and 1 h (70%) compared to the G-M dual-capped for the same reaction time. The reason for this type of behavior produced by TGA may be due to the presence of shorter carbon chain in its structure (two carbon atoms) compared to MPA (three carbon atoms) [7,12]. This property gives rise to good photo-physical attributes as mentioned in the literature and allows QDs stabilized with TGA to be used as fluorescence probes in various imaging application [12,20].

**Figure 3:**
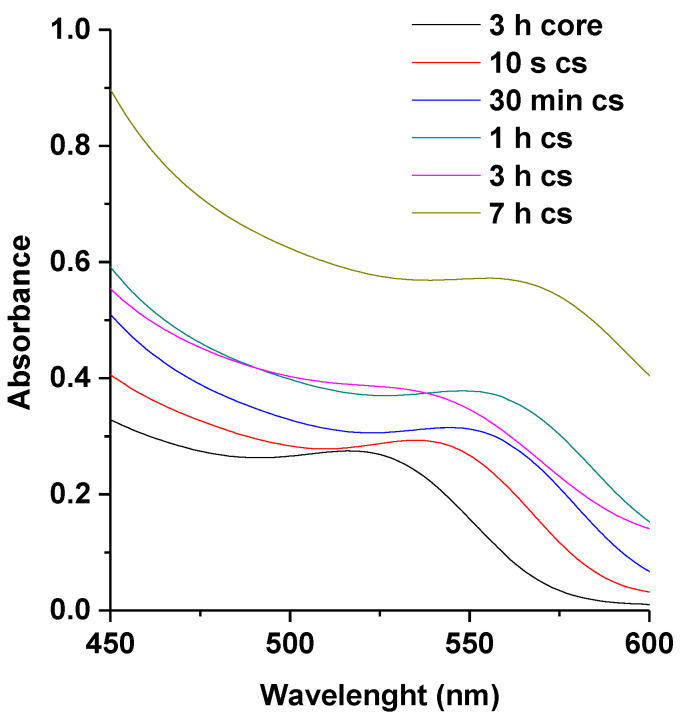
UV–VIS spectra of L-T-dual-capped CdTe cores (3 h) and CdTe/CdSe core-shells (10 s–7 h). Note: cs represent core-shell.

However, as mentioned previously in Section 3.1.1 above, the fluorescence properties of MPA-TGA (M-T) are still regarded as the best result, which is due to the observed high fluorescence intensity for all the mole ratios of Te/Se (1:0.2–1:1) for almost all reaction times compared to similar dual-capped thiols (see Supplementary Information, Figures S1–S5). In Figure 3 the UV–VIS spectra of L-T dual-capped are presented, the sharp band edges can be observed in all the reaction time, indicating a sign of good photo-physical behavior.

### 3.2. TEM Images of M-T-CdTe/CdSe Cores and Core-Shells at 3 h Reaction Time

**Figure 4:**
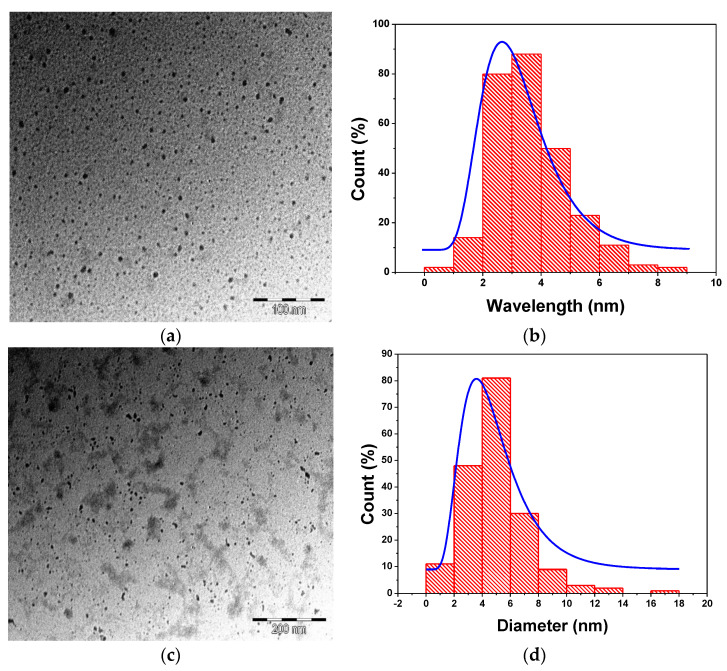
TEM image of M-T-CdTe core (**a**) and M-T-CdTe/CdSe core-shell (**c**) both at 3 h reaction time. Size distribution of the CdTe core (**b**) and CdTe/CdSe shell (**d**).

Figure 4 represents the TEM image of M-T-CdTe cores and CdTe/CdSe core-shells. It is clear from the two TEM micrograph in Figure 4a,c that there are some individual particles separated and the shape of the nanoparticle synthesized closely mimics that of QDs. The calculated average particle size for the TEM images of CdTe core in Figure 4b is 3.65 ± 1.31 while that of CdTe/CdSe core-shell in Figure 4d is 4.92 ± 2.28. The increase in average particle size from the core to the core-shell signifies that each of the core-shell particles are considerably larger than their respective cores. This is interpreted as an extra coating of the CdSe shell at the surface of the CdTe core.

### 3.3. UV and PL Spectra of MPA-TGA-CdTe/CdSe/ZnSe Core-Shell-Shell QDs

**Figure 5:**
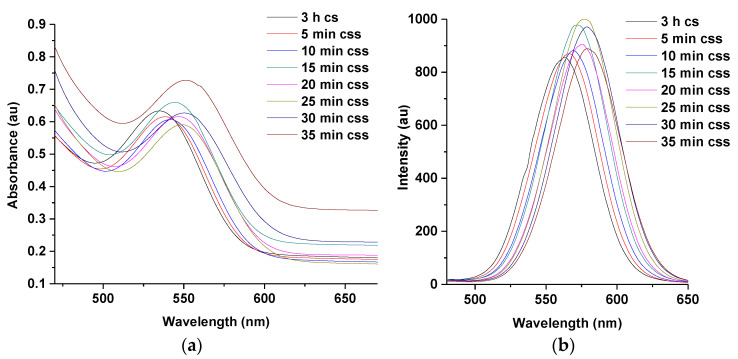
UV–VIS (**a**) and PL spectra (**b**) of MPA-TGA-CdTe core-shell-shell. Note: cs and css represent core-shell and core-shell-shell.

The PL spectra in Figure 5b demonstrate significant shift in wavelength and simultaneous increase in emission intensity at 3 h for the CdTe core shell and at 5 min for the CdTe core-shell shell following the injection of 1 mL each of Zn and Se ions, respectively. The shelling of ZnSe shell at the surface of the CdTe core-shell accounts for the increase in fluorescence intensity of the multi-core-shell. The ZnSe shell having a substantially wider band gap confines the exciton within the CdTe/CdSe interface and isolates it from the solution environment, thus leading to increase in emission intensity of the heterostructure [16,17,18,19].

The observation from the UV spectra also reveals the fabrication of CdTe multi core-shells which absorb at longer wavelength (Figure 5a). 

#### 3.3.1. PL Spectra of M-T-CdTe Multi-Core-Shells at 14 and 21 mL Injection of Zn and Se Ions

**Figure 6:**
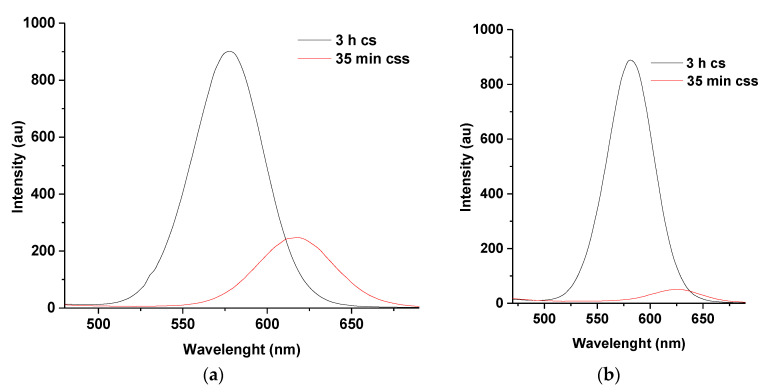

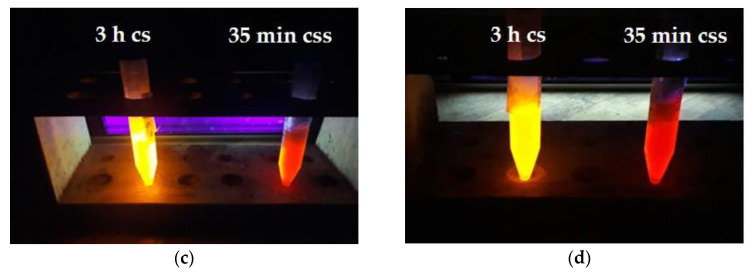
PL spectra of 3 h CdTe core-shell as compared to 35 min CdTe core-shell shell at 14 mL (**a**) and 21 mL (**b**) stabilized by MPA and TGA. (**c**,**d**) represent corresponding emission colors under UV lamp for 14 mL and 21 mL injection of Zn and Se ions. Note: ‘cs’ represents core-shell while “css” represent core-shell-shell.

Optimization of the reaction condition was also performed to improve the photo-physical properties. This was conducted by increasing the volume of Zn and Se ions to 14 and 21 mL, respectively. Injection at these volumes led to a wavelength shift of about 35 nm (14 mL) (Figure 6a) and 39 nm (21 mL) (Figure 6b) between 3 h for the CdTe core-shell and 35 min for the core-shell shell. The emission wavelength at 35 min (14 mL) is recorded at 616 nm (PLQY 30%) while that for 21 mL is observed at 625 nm (PLQY 16%). This indicates an increase in particle size due to the formation of a thicker ZnSe shell as the concentration of Zn and Se ions is increased. The significant quench in fluorescence intensity might be attributed to a large lattice mismatch as a result of the increased thickness of the ZnSe shell. This could lead to an interfacial strain at the surface of CdTe/CdSe shell and cause the trapping of photo-degenerated charge carriers. Similar observations were reported by Taniguchi et al. in 2009 and Vuyelwa et in 2011 during the synthesis of CdTe/CdSe/ZnSe core-shell-shell QDs [17,19].

The differences in wavelength shifts can further be observed in Figure 6c,d where the CdTe multi-core-shell in Figure 6d showed a stronger red emission color compared to the multi-core-shell in Figure 6c.

#### 3.3.2. PL Spectra of M-T-CdTe Multi-Core-Shell at Injection Intervals of 30 and 5 min

**Figure 7:**
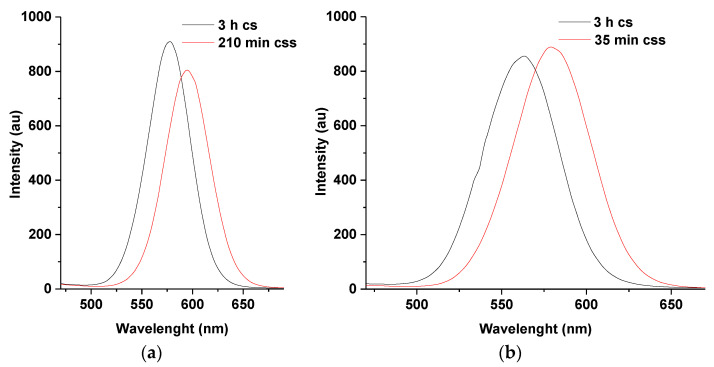
PL spectra of M-T-CdTe core-shell shell with injection of 7 mL of Zn and Se ions at 30 min (**a**) and 5 min intervals (**b**) Note: ‘cs’ represents core-shell while “css” represent core-shell-shell.

The effect of time on the wavelength shifts of the multi core-shell was also investigated by injecting 1 mL of Zn and Se ions, respectively, at 30 min intervals (Figure 7a) for over 210 min compared to the routine time intervals of 5 min discussed earlier. It was established that the wavelength shift corresponding to the 3 h core-shell and the CdTe multi-core-shell in the spectra of Figure 7a remained the same (17 nm) as the wavelength shift for the CdTe multi core-shell in spectra of Figure 7b.

This result indicates that even though the total reaction time for the fabrication of the multi core-shell in Figure 7a is 210 min, the shift in wavelength at 3 h for CdTe core-shell and at 210 min for the CdTe core-shell shell remained the same as the shift at 3 h for CdTe core-shell and the CdTe core-shell shell at 35 min as depicted in spectra of Figure 7b. Thus, it can be concluded that the relative concentrations of Zn and Se ions played a larger role in shifting the wavelength of the QDs.

#### 3.3.3. PL Emission Spectra of L-T and L-M Dual Caps Compared to G-T and G-M at 7 mL Injection of Zn and Se Ions

**Figure 8:**
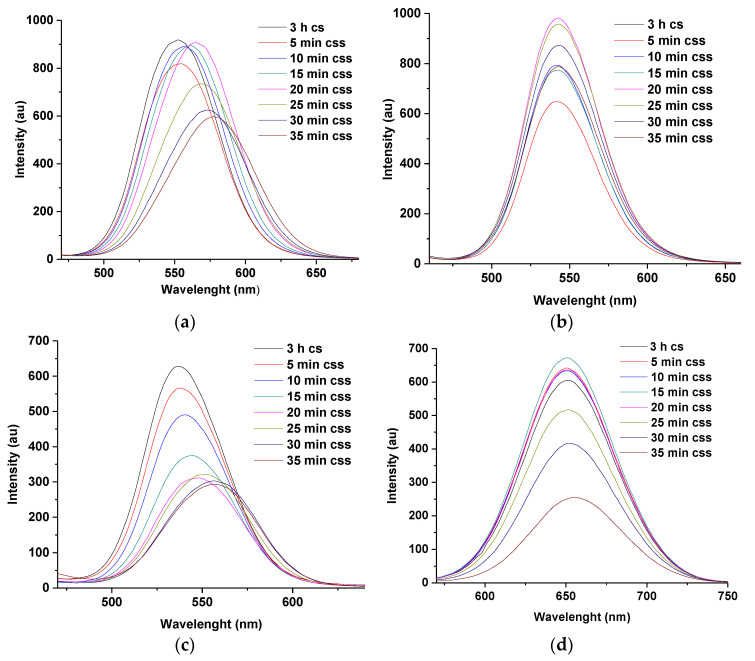
PL emission spectra of L-T (**a**) and L-M (**c**) dual caps compared to G-T (**b**) and G-M (**d**) using 7 mL of Zn and Se ions at 3 h cs–35 min css. Note ‘cs’ means core-shell and ‘css’ means core-shell shell.

Following the same procedure used to synthesize M-T dual-capped CdTe multi core-shell discussed earlier, the other four CdTe core-shells containing various thiol dual caps discussed in Section 3.1.3 were also promoted to their corresponding multi-core-shells resulting in improved photo physical properties. However, it was observed that multi-core shells dual-capped with L-M and L-T demonstrated similar fluorescent behaviors to M-T-CdTe core-shell-shell compared to G-T and G-M dual caps. The similarities of the fluorescence spectra for these three (M-T, L-T and L-M duals caps) is mainly due to similarities of their corresponding wavelength shifts as a consequence of the formation of the ZnSe shell at the surface of CdTe/CdSe core-shell by addition of 7 mL of Zn and Se ions, respectively. This behavior was absent for G-T and G-M dual caps. The corresponding spectra in Figure 8a–d illustrated this point by comparing the PL spectra of L-T and L-M-dual-capped (Figure 8a,c)to those for G-T and G-M dual-capped (Figure 8b,d). The lack of any wavelength shift in florescence wave-length of G-T and G-M dual cap can also be inferred from the calculated particle sizes in Table 2 compared to the particle size of M-T, L-T, and L-M. The only change in G-M and G-T dual caps is a gradual enhancement of emission intensity during the formation of ZnSe shells indicating an increment in surface passivation of the synthesized material.

**Figure 9:**
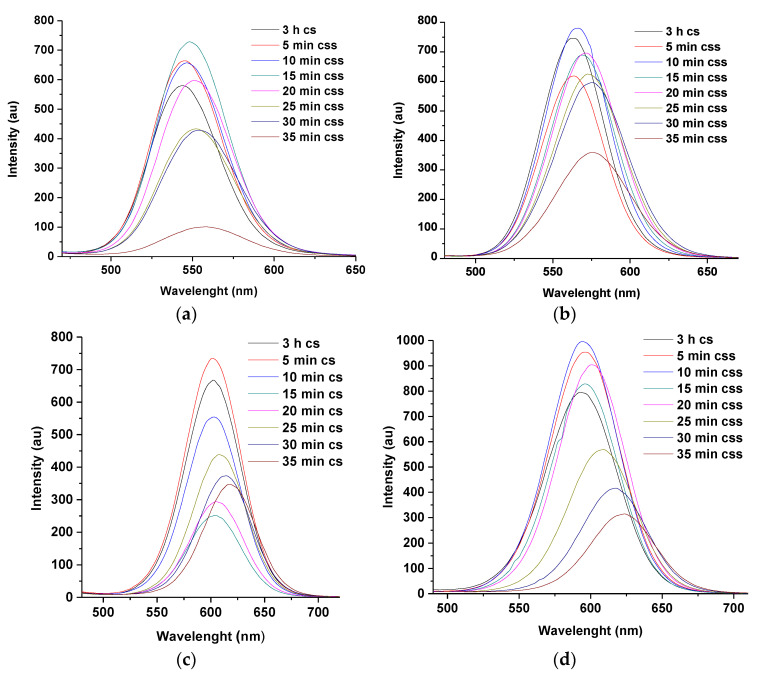
PL emission spectra of G-T (**a**,**b**) and G-M (**c**,**d**) dual-capped CdTe css compared with 14 and 21 mL of Zn and Se ions at 3 h cs–35 min css. Note ‘cs’ means core-shell and ‘css’ means core-shell shell.

It is possible that, the presence of more capping groups (carboxylate and amino) in the structure of glutathione (GSH) might be responsible for the absence of shift in the emission wavelength of G-T and G-M dual following injection of 7 mL each of Zn and Se ions into the reacting mixture. The observation is further highlighted through Figure 8b, that is, as the shell growth increases, the fluorescence intensity of G-T-CdTe css at 25 and 30 min seems to be higher than those of G-M-CdTe css at similar reaction times (Figure 8d). Similar observation can be made with L-cysteine-TGA (Figure 8a) compared to L-cysteine-MPA (Figure 8c) at the same reaction times. These observations were also noticed during the course of the synthesis of their corresponding core-shells as previously discussed in Figure 2. All these observations point towards the positive effects of using TGA as a dual stabilizing agent [3,7,12,20,21]. Moreover, when the reaction conditions for the two dual-capped were changed to 14 and 21 mL of Zn and Se ions, respectively, some shift in emission wavelength was observed after 10 min of reaction time, this gradually increased as reaction approached 35 min (Figure 9a–d). It was further discovered from the study of the two dual caps that injection of 21 mL of Zn and Se ions gave the highest wavelength shift (13 nm for G-T and 30 nm for G-M) at 3 h for the core-shell and 35 min for the core-shell shell. Furthermore, the maxima for emission wavelengths at 35 min for both G-T and G-M dual caps can be found at 576 nm and 620 nm, respectively.

Furthermore, in G-T dual-capped PL spectra (14 mL) (Figure 9a), the emission intensity increased slightly at 5–20 min for css compared to 3 h for cs. This indicates passivation provided by the ZnSe shell at the surface of CdTe/CdSe core-shell. Furthermore, the observed fluctuations in emission intensities observed for some reactions times may be a result of competition between stability and luminescence in aqueous media [13]. Nevertheless, the capping provided by most of the thiol stabilizing agents discussed here was significant with glutathione demonstrating enhanced capping functionality, this may be ascribed to the presence of more coordinating sites in its structure [13].

**Figure 10:**
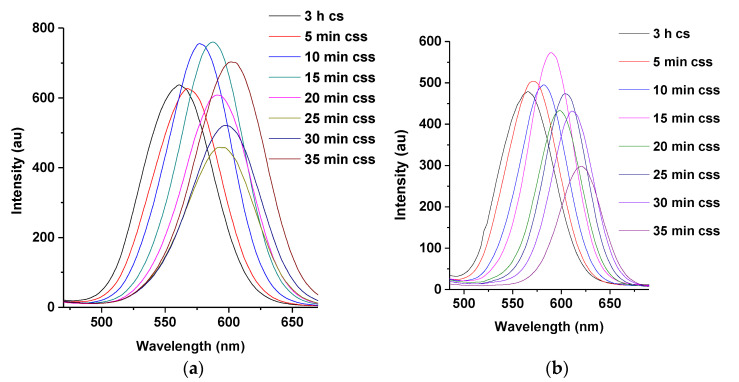
PL emission spectra of L-T (**a**) and L-M (**b**) dual-capped CdTe css at 21 mL injection of Zn and Se ions at 3 h cs–35 min css. Note ‘cs’ means core-shell and ‘css’ means core-shell shell.

The optimization of reaction condition conducted for L-T and L-M dual-capped using 21 mL of Zn and Se ions, respectively, is observed in Figure 10a,b. The emission maxima at 35 min reaction time can be observed at 602 nm and 620 nm, respectively. These studies confirm that MPA-TGA-CdTe css demonstrated the highest emission maxima of 625 nm at 35 min reaction time following the injection of 21 mL of Zn and Se ions, respectively.

### 3.4. TEM Images of CdTe/CdSe/ZnSe Core-Shell-Shell

**Figure 11:**
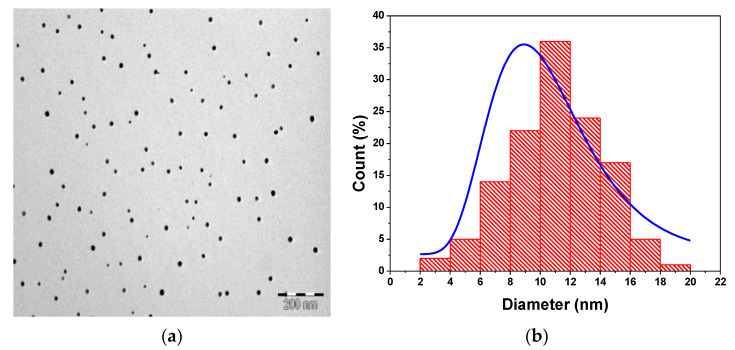
TEM image of M-T CdTe/CdSe/ZnSe multi-core-shell QDs (**a**) with its corresponding particle size distribution (**b**).

The TEM morphology for M-T-CdTe core-shell-shell at 35 min reaction time (21 mL of Zn and Se ion injection) is represented in Figure 11a. The formation of a spherical shape nanoparticle can be observed as we reported in the TEM image for their corresponding cores and core-shell displays in Figure 4a,c. The average particle size from the TEM image is calculated to be 10.5 ± 3.16, which is larger than those of the core-shell discussed earlier (Figure 4d). This reveals that each multi-core-shell particle is considerably larger in size than its core-shell counterpart, and this could be attributed to the formation of a thicker ZnSe shell at the surface of CdTe/CdSe core-shell. The injection of the top volume of 21 mL of Zn and Se ions might be a contributing factor for the larger particle size.

In addition, the FTIR spectra of L-T-CdTe/CdSe core-shell QDs and the EDS spectrum of M-T-CdTe/CdSe/ZnSe core-shell-shell QDs are shown in Supplementary Information Figures S6 and S7.

### 3.5. Exposure of M-T-CdTe/CdSe Core-Shell and Multi-Core-Shell to UV Illumination

**Figure 12:**
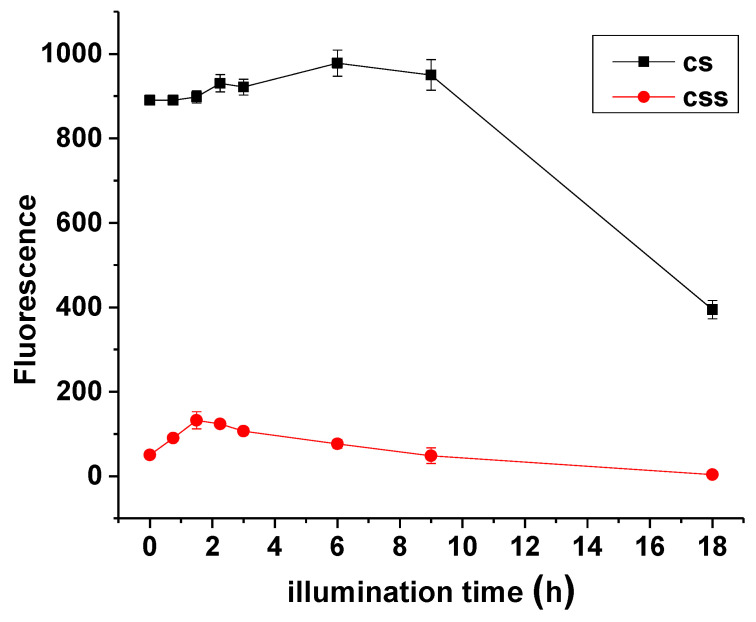
M-T-CdTe/CdSe core-shell (3 h) and CdTe/CdSe/ZnSe core-shell-shell (35 min) exposed to continuous UV irradiation for 18 h.

Figure 12 illustrates the photostability investigation of CdTe/CdSe core-shell QDs at 3 h reaction time and CdTe/CdSe/ZnSe multi-core-shell at 35 min reaction time under continuous exposure to UV radiation for 18 h. It is clear that as the illumination time increases, the two QDs demonstrate an enhancement in fluorescence with the core-shell having the highest fluorescence intensity at PLQY of 89% corresponding to a 6 h illumination time, while the multi-core-shell demonstrates its highest fluorescence intensity at PLQY of 23% corresponding to a 1.5 h illumination time. Therefore, the PLQY for the core-shell has increased by 16% from the initial values of 73% while the multi-core-shell has increased by 7% from the initial values of 16%. Similar demonstrations that have been reported in some literature on the effects of UV light on QDs, revealing that the increase in fluorescence intensity could be due to photoetching effects and surface re-combination processes during exposure of UV radiation to QD surfaces [25,26,27]. Similar observation was also reported by Hosnedlova et al. in 2020 during their study of physico-chemical changes of CdTe QDs after their exposure to environmental conditions [28]. Furthermore, it is observed that, as the illumination time increases, the two QDs show some florescence quenching which leads to a significant decrease in their corresponding PLQY values.

Gradual decreases in fluorescence for the multi-core-shell was observed from 2.25 h illumination time which proceeded to deteriorate drastically up to 18 h of exposure time to UV irradiation. The sharp decline in florescence of CdTe/CdSe core-shell was also observed from 9 h to 18 h of illumination time. As reported from previous studies, prolonged exposure of QDs to UV irradiation causes the release of metal atoms from the surface due to the oxidation process. This observation was reported by Derfus et al. during the exposure of CdSe QDs to UV radiation [29]. It was further explained by Hosnedlova et al. that, during this oxidation process, the O_2_ molecules oxidize chalcogenide atoms located on the surface of the QDs to form oxides [28]. These oxide molecules desorb from the surface, leaving behind “dangling” reduced metallic atoms. Hence, in our case, prolonged exposure of these QDs to an oxidative environment might have led to the decomposition of their structure, thereby leading to desorption of Cd^2+^ ions or Zn^2+^ from the CdTe/CdSe or CdTe/CdSe/ZnSe complexes. Furthermore, we also assumed that the slight percentage increase in the PLQY of CdTe multi-core-shell QDs compared to its corresponding core-shell might be due to the large lattice mismatch between the interface of the CdSe and ZnSe shell as a result of the formation of a thick ZnSe shell at a 21 mL injection of Zn and Se ions. This factor might also be responsible for the quick loss in fluorescence of the nanoparticles upon further exposure to UV illumination. However, further studies are being contemplated to verify the role of some of these factors.

## 4. Conclusions

In this paper we reported the synthesis of five thiol dual-capped CdTe core-shells using MPA-TGA, GSH-MPA, GSH-TGA, L-cysteine-TGA, and L-cysteine-MPA all showing improved photo-physical properties. The optimization of the reaction condition was achieved by varying the concentration of reduced selenium into the reaction vessels to give various mole ratios of Te:Se from 1:0.2 to 1:1, respectively. However, it was observed that almost all the CdTe dual-capped core-shells showed their highest emission intensities at 3 h (1:0.2 of Te:Se) compared to other mole ratios for equal reaction times. Therefore, the fabrication of all the dual-capped CdTe multi-core shells were based on this reaction time (3 h) using 1:0.2 of Te:Se with the exception of MPA-TGA, where its 3 h reaction time showed high intensities throughout the optimized reaction conditions. It was also observed that the use of TGA as one of the dual stabilizing agents in the case of GSH-TGA, L-cysteine-TGA, and MPA-TGA demonstrated enhanced fluorescence compared to GSH-MPA and L-cysteine-MPA. Nevertheless, the combination of MPA-TGA to stabilize the surface of the QDs was observed to produce higher fluorescence intensities at all mole ratio of Te/Se and this can be seen virtually in all its reaction times compared to the other four remaining dual-capped thiols. 

Furthermore, the synthesis of five dual-capped multi core-shells with enhanced optical properties was achieved with rather shorter reaction time of 5 min and maximum time of 35 min. Upon optimization of reaction conditions, the largest shift in wavelength at 3 h for the core-shell and 35 min for the multi-core-shell corresponded to the 21 mL injection of Zn and Se ions, respectively. The change in injection time intervals from 5 min to 30 min contributed no significant differences in wavelength shift compared to the shift caused by variation in the amount of Zn and Se ion. It was further observed that multi-core-shell dual-capped with glutathione (GSH) demonstrated a significant shift in wavelength only after 14- and 21 mL injection of Zn and Se ions, respectively. Injections at 7 mL of Zn and Se ions only enhanced their fluorescence intensity with no significant shift in their wavelength. The positive effects of using TGA as a co-stabilizing agent was also observed during the shell growth from 25–35 min by comparing the fluorescence intensity of GSH-TGA-CdTe css to GSH-MPA-CdTe css. The same observation can be made with L-cysteine-TGA-CdTe css compared to L-cysteine-MPA-CdTe css at a similar reaction time. The main observation is that, for all the thiol-dual caps, the highest emission maxima occur at a 35 min reaction time corresponding to a 21 mL injection of Zn and Se ions, for MPA-TGA dual cap (PLQY 16%). A further fluorescence enhancement with PLQY of 23% was observed during the continuous exposure of MPA-TGA-CdTe multi-core-shell QDs at 35 min reaction times to UV radiation for 18 h. Similarly, the exposure of MPA-TGA-CdTe core-shell QDs to UV radiation at 3 h reaction time led to an increase in its PLQY from 73% to 89%. The EDS result also confirmed the fabrication of CdTe multi core-shell by revealing the presence of Zn and Se ions. It can be concluded that the synthetic approach followed in this study can be used to produce more CdTe multi-core-shell heterostructures at longer emission wavelengths, this can be achieved within a short reaction time. The use of dual capping thiol especially TGA to stabilize the surface of synthesized core-shell and multi-core-shell QDs also assisted in the improvement of the surface chemistry of the synthesized chalcogenides.

## Figures and Tables

**Table 1:** Estimated particle size of CdTe cores (3 h) and CdTe/CdSe core-shells (50 s–7 h) for M-T at 1:1, Te/Se and (10 s–7 h) for G-M, G-T, L-T, and L-M at 1:0.2, Te/Se).

Reaction Time	M-T (nm)	Reaction Time	G-M (nm)	G-T (nm)	L-T (nm)	L-M (nm)
3 h core	2.7	3 h core	3.26	1.9	2.6	2.7
50 s	2.9	10 s	3.27	2.1	2.7	2.9
30 min cs ^1^	3.1	30 min	3.31	2.2	3.1	3
1 h cs	3.2	1 h cs	3.33	2.3	3.2	3.1
3 h cs	3.4	3 h cs	3.34	2.6	3.3	3.2
7 h cs	3.5	7 h cs	3.35	3.3	3.4	3.6

^1^ core-shell.

**Table 2:** Estimated particle size of CdTe cs (3 h) and CdTe/CdSe/ZnSe core-shell-shells (5 min–35 min) for M-T, G-M, G-T, L-T, and L-M dual caps at injections of 7 mL each of Zn and Se ions.

Reaction Time	M-T (nm)	G-M (nm)	G-T (nm)	L-T (nm)	L-M (nm)
3 h cs ^1^	2.9	3.5	2.4	2.4	2.5
5 min css ^2^	3.12	3.5	2.4	2.5	2.6
10 min css	3.14	3.5	2.4	2.6	2.7
15 min css	3.16	3.5	2.4	2.7	2.9
20 min css	3.19	3.5	2.4	2.8	3.1
25 min css	3.22	3.5	2.4	2.9	3.3
30 min css	3.24	3.5	2.4	3	3.5
35 min css	3.25	3.6	2.5	3.2	3.6

^1^ core-shell and ^2^ core-shell-shell.

**Table 3:** Estimated particle size of CdTe cs (3 h) and CdTe/CdSe/ZnSe core-shell-shell (5 min–35 min) for M-T, G-M, G-T, L-T, and L-M dual caps at injections of 14 mL each of Zn and Se ions.

Reaction Time	M-T (nm)	G-M (nm)	G-T (nm)	L-T (nm)	L-M (nm)
3 h cs ^1^	3.2	3.43	2	2.3	2
5 min css ^2^	3.3	3.43	2.1	2.6	2.1
10 min css	3.32	3.44	2.13	2.8	2.13
15 min css	3.34	3.45	2.14	2.9	2.15
20 min css	3.37	3.46	2.16	3.0	2.17
25 min css	3.4	3.48	2.17	3.2	2.2
30 min css	3.44	3.5	2.18	3.3	2.25
35 min css	3.47	3.55	2.2	3.4	2.28

^1^ core-shell and ^2^ core-shell-shell.

**Table 4:** Estimated particle size of CdTe cs (3 h) and CdTe/CdSe/ZnSe core-shell-shell (5 min–35 min) for M-T, G-M, G-T, L-T, and L-M dual caps at injections of 21 mL each of Zn and Se ions.

Reaction Time	M-T (nm)	G-M (nm)	G-T (nm)	L-T (nm)	L-M (nm)
3 h cs ^1^	3.3	2.44	2.19	2.6	2.3
5 min css ^2^	3.33	2.46	2.19	2.8	2.4
10 min css	3.35	2.47	2.2	3.0	2.6
15 min css	3.37	2.48	2.24	3.1	2.8
20 min css	3.38	2.5	2.26	3.2	3.0
25 min css	3.39	2.56	2.27	3.3	3.3
30 min css	3.47	2.62	2.29	3.4	3.6
35 min css	3.48	2.7	2.3	3.5	3.8

^1^ core-shell and ^2^ core-shell-shell.

## Data Availability

The data presented in this study are available on request from the corresponding author.

## Supplementary Materials

The following are available online at https://www.mdpi.com/article/10.3390/nano11051193/s1. Figure S1: Emission spectra of M-T-CdTe/CdSe core shells at various mole ratios of Te/Se: A (1:0.2), B (1:0.4), C (1:0.6), D (1:0.8) and E (1:1). Figure S2: Emission spectra of G-M-CdTe/CdSe core shells at various mole ratios of Te/Se: A (1:0.2), B (1:0.4), C (1:0.6), D (1:0.8) and E (1:1). Figure S3: Emission spectra of G-T-CdTe/CdSe core shells at various mole ratios of Te/Se: A (1:0.2), B (1:0.4), C (1:0.6), D (1:0.8) and E (1:1). Figure S4: Emission spectra of L-T-CdTe/CdSe core shells at various mole ratios of Te/Se: A (1:0.2), B (1:0.4), C (1:0.6), D (1:0.8) and E (1:1). Figure S5: Emission spectra of L-M-CdTe/CdSe core shells at various mole ratios of Te/Se: A (1:0.2), B (1:0.4), C (1:0.6), D (1:0.8) and E (1:1). Figure S6: FTIR spectra of L-T-CdTe/CdSe core-shell. Figure S7: EDS analysis of M-T dual capped multi-core-shell.
