# Supplementary material for: Rapid Synthesis of Thiol-Co-Capped-CdTe/CdSe/ZnSe Core Shell-Shell Nanoparticles: Their Optical and Structural Morphology
Source: Nanomaterials (Basel). 2021 May 1;11(5):1193. doi: 10.3390/nano11051193 (PMC8147246; doi:10.3390/nano11051193)
Supplement: Supplementary file 1 [file nanomaterials-11-01193-s001.zip › nanomaterials-1159291-supplementary.pdf]

Supplementary information

Rapid Synthesis of Thiol-Co-Capped-CdTe/CdSe/ZnSe Core Shell-Shell Nanoparticles: Their Optical and Structural Morphology

Olamide Abiodun Daramola [1], Xavier Siwe-Noundou [1,*], Potlaki Foster Tseki [2] and Rui Werner Maçedo Krause [1,*]

[1] Department of Chemistry, Faculty of Science, Rhodes University, P.O. Box 94, Grahamstown 6140, South Africa; truelamm@gmail.com
[2] Department of Chemical and Physical Sciences, Faculty of Natural Science, Walter Sisulu University, Private Bag XI, Mthatha 5117, South Africa; ptseki@wsu.ac.za
* Correspondence: x.siwenoundou@ru.ac.za (X.S.-N.); r.krause@ru.ac.za (R.W.M.K.), Tel.: +27-46-603-7030 (X.S.-N. & R.W.M.K.)

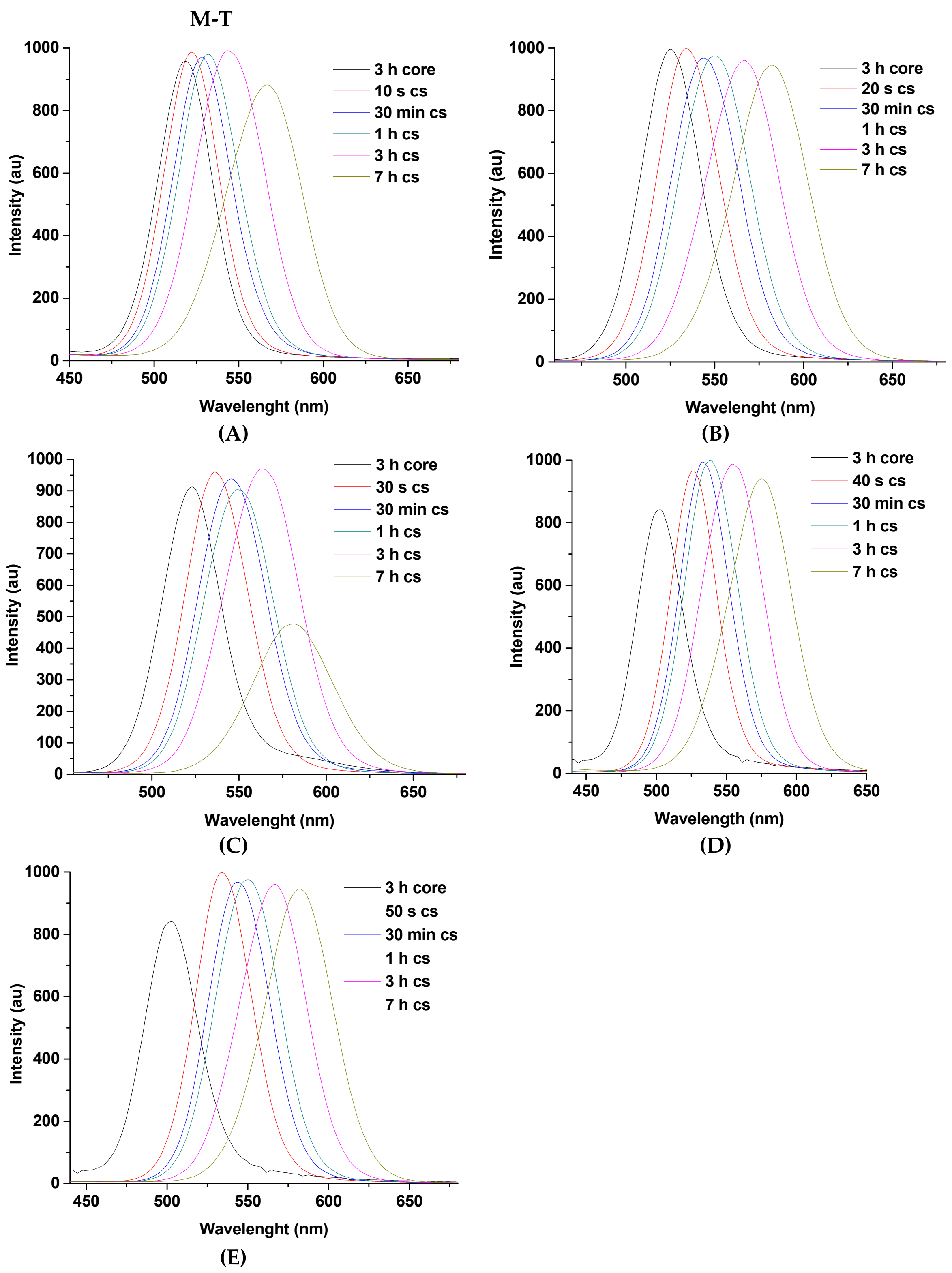

Figure S1. Emission spectra of M-T-CdTe/CdSe core shells at various mole ratios of Te/Se: (**A**) (1:0.2), (**B**) (1:0.4), (**C**) (1:0.6), (**D**) (1:0.8) and (**E**) (1:1). Note: 'CS' represent core–shell.

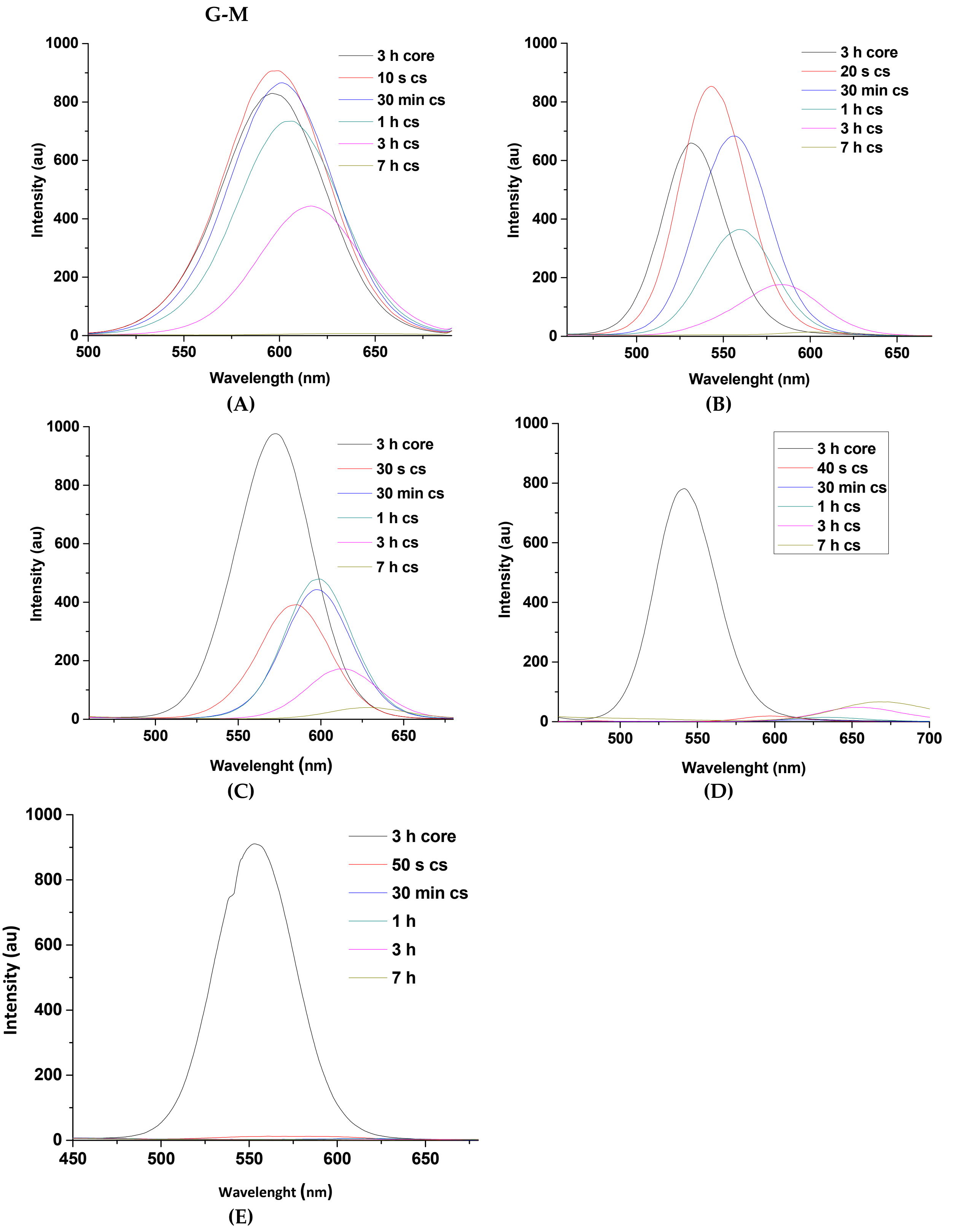

Figure S2. Emission spectra of G-M-CdTe/CdSe core shells at various mole ratios of Te/Se: (**A**) (1:0.2), (**B**) (1:0.4), (**C**) (1:0.6), (**D**) (1:0.8) and (**E**) (1:1). Note: 'CS' represent core–shell.

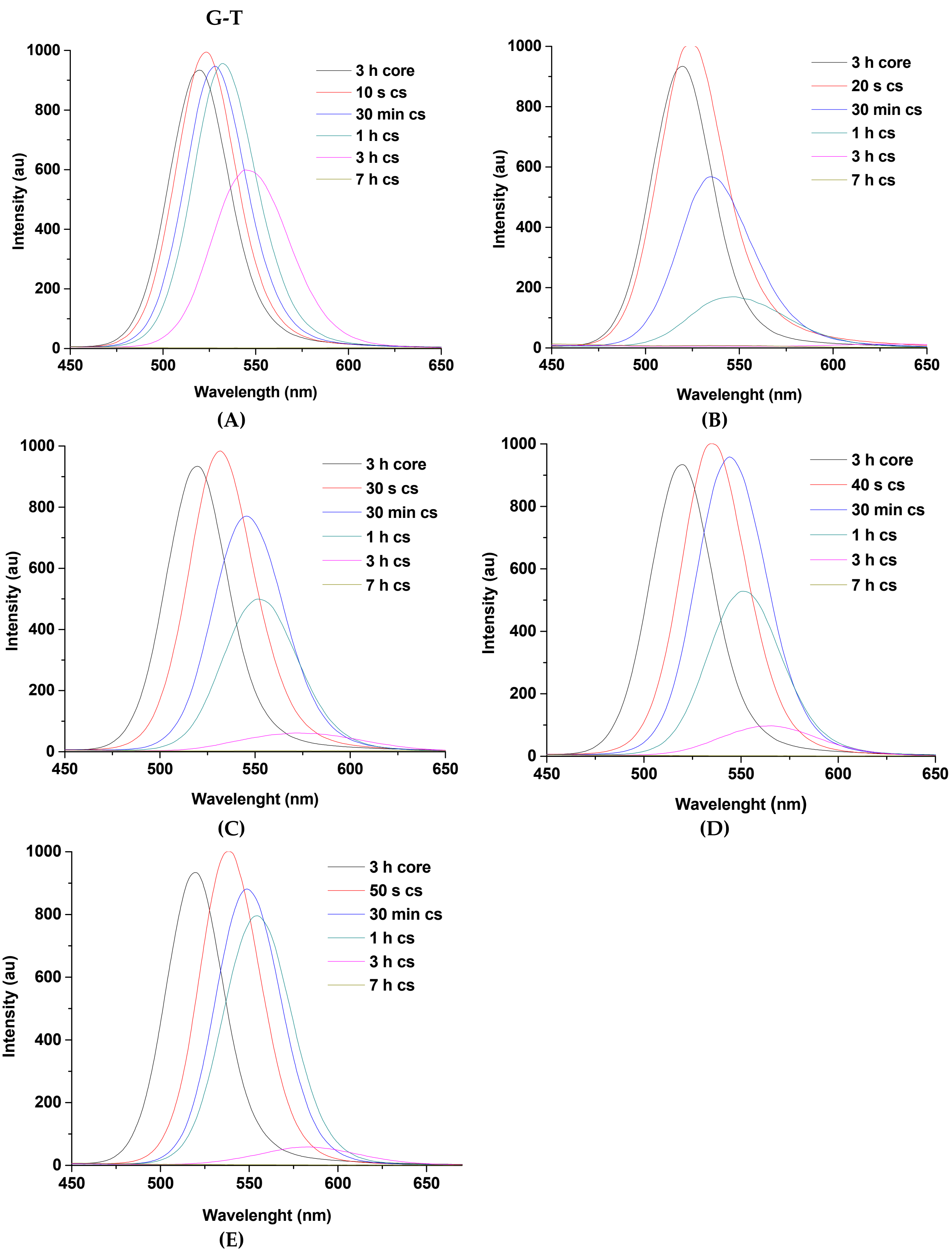

Figure S3. Emission spectra of G-T-CdTe/CdSe core shells at various mole ratios of Te/Se: (**A**) (1:0.2), (**B**) (1:0.4), (**C**) (1:0.6), (**D**) (1:0.8) and (**E**) (1:1). Note: 'CS' represent core–shell.

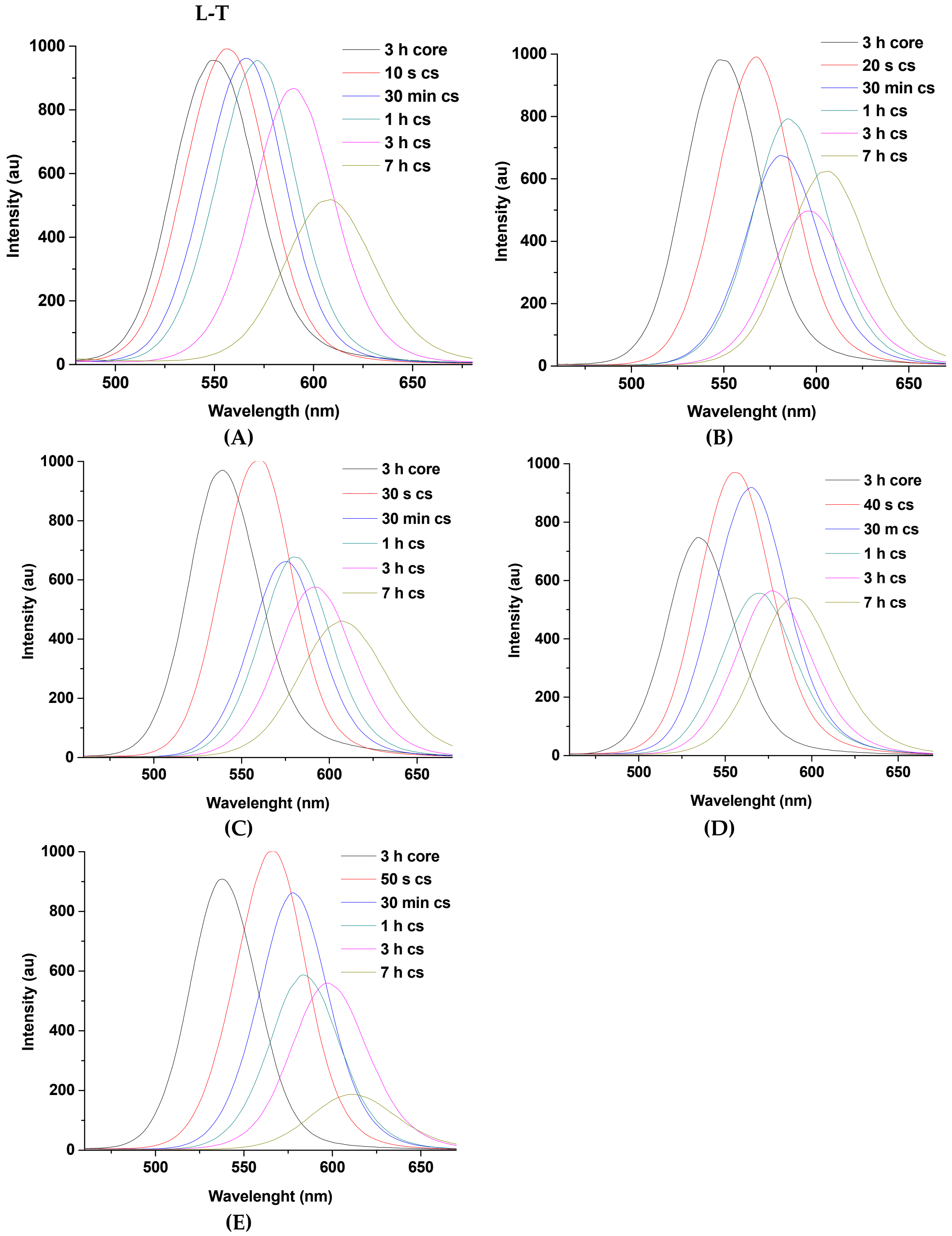

Figure S4. Emission spectra of L-T-CdTe/CdSe core shells at various mole ratios of Te/Se: (**A**) (1:0.2), (**B**) (1:0.4), (**C**) (1:0.6), (**D**) (1:0.8) and (**E**) (1:1). Note: 'CS' represent core–shell.

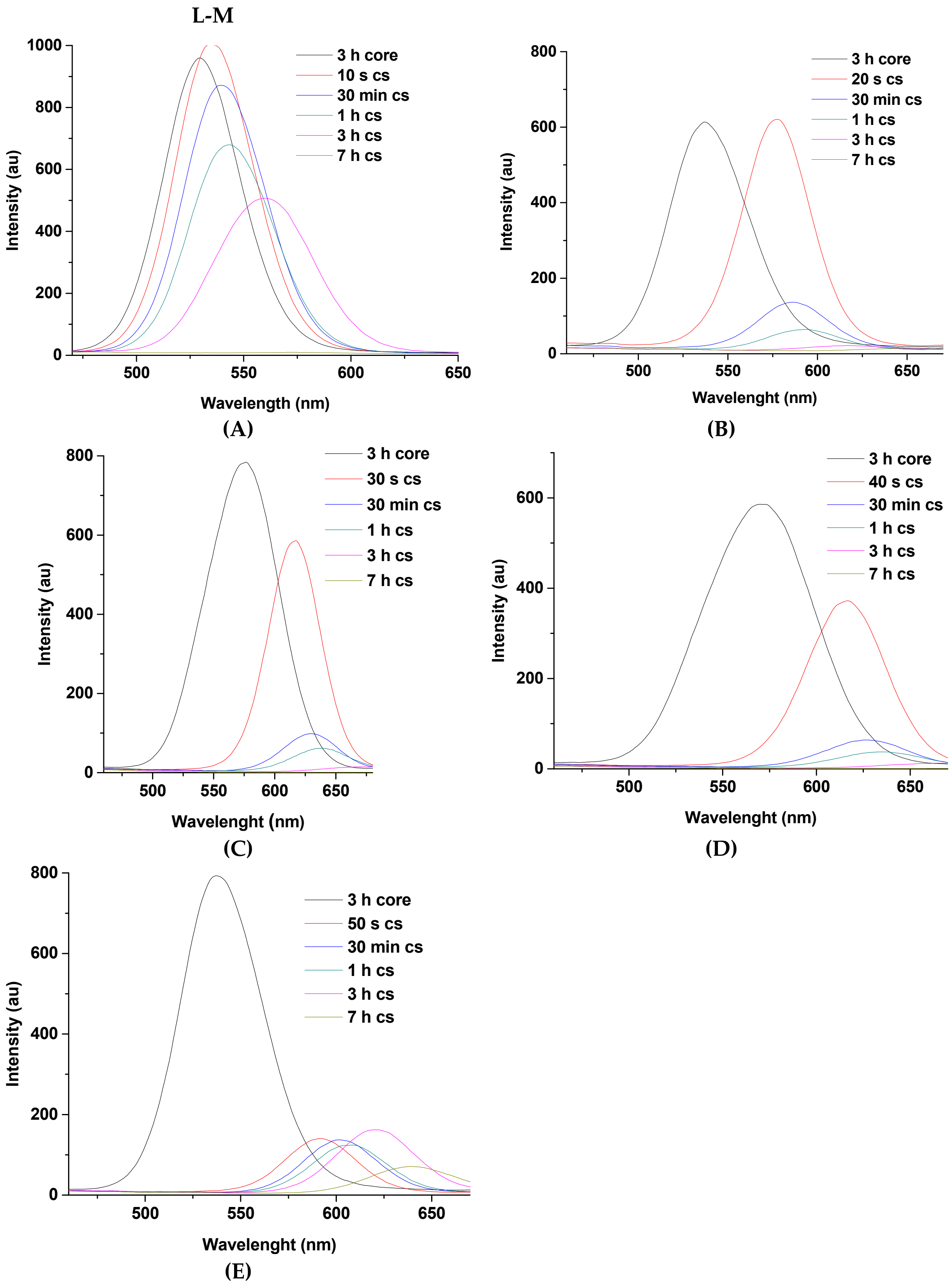

Figure S5. Emission spectra of L-M-CdTe/CdSe core shells at various mole ratios of Te/Se: (**A**) (1:0.2), (**B**) (1:0.4), (**C**) (1:0.6), (**D**) (1:0.8) and (**E**) (1:1). Note: 'CS' represent core–shell.

FTIR spectra of L-T CdTe core-shell QDs

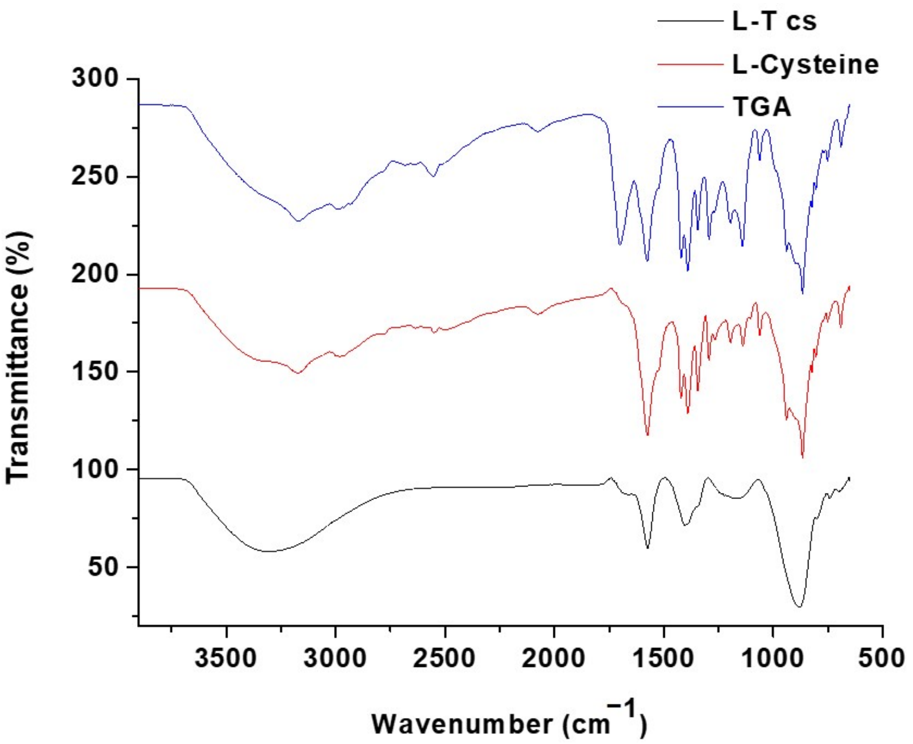

Figure 6. FTIR spectra for L-T-CdTe/CdSe core-shell.

EDS analysis

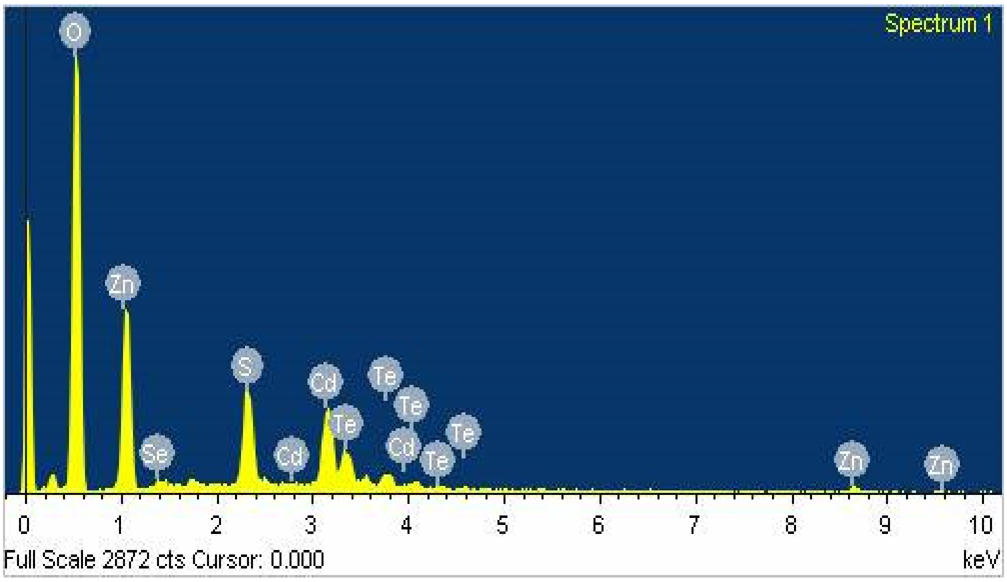

Figure S7. EDS analysis of M-T dual capped multi core-shell.
